# Setting health care services tariffs in Iran: half a century quest for a window of opportunity

**DOI:** 10.1186/s12939-020-01224-1

**Published:** 2020-07-06

**Authors:** Leila Doshmangir, Arash Rashidian, Farhad Kouhi, Vladimir Sergeevich Gordeev

**Affiliations:** 1 Tabriz Health Services Management Research Center, Iranian Center of Excellence in Health Management, School of Management and Medical Informatics, Tabriz University of Medical Sciences, Tabriz, Iran; 2 Social Determinants of Health Research Centre, Health Management and Safety Promotion Research Institute, Tabriz University of Medical Sciences, Tabriz, Iran; 3 Department of Health Policy & Management, School of Management and Medical Informatics, Tabriz University of Medical Sciences, Tabriz, Iran; 4 Department of Science, Information and Dissemination, World Health Organization Regional Office for the Eastern Mediterranean, Cairo, Egypt; 5 Department of Health Management and Economics, School of Public Health, Tehran University of Medical Sciences, Tehran, Iran; 6 Treatment Deputy, Iran Social Security Organization, Tehran, Iran; 7 Institute of Population Health Sciences, Queen Mary University of London, London, UK; 8 Department of Infectious Disease Epidemiology, London School of Hygiene & Tropical Medicine, London, UK

**Keywords:** Policy-making, Medical tariff, Medical payment, Medical pricing, Health policy and system research, Policy triangle framework, Iran

## Abstract

**Background:**

The process of medical tariffs setting in Iran remains to be a contentious issue and is heavily criticized by many stakeholders. This paper explores the experience of setting health care services tariffs in the Iranian health care system over the last five decades.

**Methods:**

We analyzed data collected through literature review and reviews of the official documents developed at the various levels of the Iranian health system using inductive and deductive content analysis. Twenty-two face-to-face semi-structured interviews supplemented the analysis. Data were analysed and interpreted using ‘policy triangle’ and ‘garbage can’ models.

**Results:**

Our comprehensive review of changes in the medical tariff setting provides valuable lessons for major stakeholders. Most changes were implemented in a sporadic, inadequate, and a non-evidence-based manner. Disparities in tariffs between public and private sectors continue to exist. Lack of clarity in tariffs setting mechanisms and its process makes negotiations between various stakeholders difficult and can potentially become a source of a corrupt income. Such clarity can be achieved by using fair and technically sound tariffs. Technical aspects of tariff setting should be separated from the political negotiations over the overall payment to the medical professionals. Transparency regarding a conflict of interest and establishing punitive measures against those violating the rules could help improving trust in the doctor-patient relationship.

**Conclusion:**

Use of evidence-informed models and methods in medical tariff setting could help to strike the right balance in the process of health care services provision to address health system objectives. A sensitive application of policy models can offer significant insights into the nature of medical tariff setting and highlight existing constraints and opportunities. This study generates lessons learned in tariffs setting, particularly for low- and middle-income countries.

## Introduction

To prevent healthcare market failures due to possible externalities, failures of competition and market controller information asymmetry, governments may decide to intervene directly or indirectly by setting medical tariffs (also termed as prices, fees, or rates) for goods and services and introducing price ceilings and floors [1, 2]. Medical tariffs usually include a set of prices reimbursed for provided health care goods and services and payment rules for purchasers and providers valid for a specified period. Being an essential component of the broader activity of resource allocation and purchasing in any healthcare system, setting tariffs can be used to regulate the relationship between key healthcare stakeholders (i.e., providers, recipients, payers, and purchasers), determine the content of the benefits package, and guide purchasing decisions within the overall financing system [3, 4]. Medical tariffs can be applied to any type of health care service but will correspond to existing service coverage and resource distribution in public and private healthcare sectors. They are also central to establishing sound payment systems for public health and healthcare services. In many countries such as Australia, England, France, Germany, and Japan, tariff setting is integrated into provider payment systems.

Factors that influence tariffs setting include the total public spending on health, costs of service delivery, wages for specialists and other health workers, as well as the burden of specific diseases [4]. Tariffs setting also depend on the existing healthcare sector’s financial strategy and may impact the financial access to health services through patients’ out-of-pocket expenses, as well as the availability of medical services [5, 6]. Political context and existing health care policies, country’s high-level economic indicators (such as public sector expenditure and debt, household expenditure and inflation), social context (including religious and cultural beliefs), health sector and general regulatory power of the public sector (to implement the set prices) would also contribute towards determining the final medical tariffs.

Setting the right medical tariffs can help to strike a balance in the healthcare market, fulfil society’s healthcare needs, provide maximum efficiency in resource allocation and consumption, incentivize health care providers to deliver services in line with policy goals. For example, it can be used as a policy tool to provide incentives and influence provider behaviours to encourage, demarcate or limit the provision of certain services (e.g., end-of-life care at the patient’s home, long-term care and community care) or determine the geographical distribution of health care providers by raising and lowering the tariffs [7–10]. In many countries, medical tariffs are used as one of the essential tools by policymakers to influence equity, efficiency, quality, responsiveness, and accessibility to health services [11].

Mechanisms and policy strategies to determine resulting medical tariffs vary. For example, in Italy, since 1994, health care tariffs are used to regulate the healthcare system and reduce direct government involvement in controlling public providers [12]. In England, the Payment by Results system (introduced in 2003/04) is used to pay healthcare providers a standard national tariff for each patient seen or treated [13]. In the US, in the 1970s, Resource-Based Relative Value Units represented a new way to define the number of physician services and their pricing [14, 15]. In France, by 2001, negotiation between health insurance funds and union representatives for each profession was an essential step in reaching the unit value that was applied to the tariff to determine the price reimbursed for each procedure [16]. In Iran, a national system of medical tariff setting for provider reimbursement is used since 1972; however, its tariff setting process remains to be a contentious issue and is heavily criticized by various stakeholders [11, 17]. For example, there is a significant difference in medical tariffs for similar services between the public and private sectors. This difference also led to a substantial income discrepancy and is one of the main motivating factors for working in the private sector and developing dual practices.

Overall, the history of reforms that led to the current medical tariffs system in Iran is complex, and, to the best of our knowledge, no previous study has examined in detail the complexity of changes in the medical tariffs system in terms of mechanisms, governance, and shared-decision making. Given continuous pleas by various stakeholders to change the process of medical tariffs setting and calls to use an evidence-based approach upon future revisions [11, 18], the aim of this paper is two-fold: (i) to document the complexity of the development of the medical tariffs setting process; and (ii) to identify major shortfalls and drawback of the tariffs implementation process and suitable ways forward.

### Healthcare system financing background in Iran

The Iranian healthcare system is a public-private partnership. Public hospitals have two primary funding sources: the government via a line-item budget (through the Ministry of Health and Medical Education (MoHME) and medical universities) and reimbursement by the Social Security Organization (SSO) s and the Iran Health Insurance Organization (IHIO), based on fee-for-service (FFS) and per-diem payments [19]. Additionally, patients should pay 10% towards the cost of hospitalization in public hospitals, 30% for outpatient services, 25% for para-clinical services, and 20% for non-para clinical services as a co-payment. In the private sector, where tariffs are typically higher, the markup difference from the public sector tariffs is covered by a patient’s co-payment. The line-item budgets include salaries for physicians and other staff, as well as medical equipment, based on the national salary grid. FFS payments are linked to the national medical tariffs (hereafter, the tariffs) for the health care services that each physician provides and are used as an incentive for further provision of care in public hospitals [20]. Private hospitals revenues are almost entirely based on these tariffs, paid as FFS to the hospitals by the MoHME. Private hospitals use the same tariffs structure to pay physicians that provide care, although they deduct overheads and running costs from the tariff rates received [21]. Basic and supplementary insurance funds, as well as OOPs, fund private health care services, while the provision of the health care services is based on FFS payments.

The MoHME at the national level and medical at the national level and medical universities at the regional level are governing and steering the development of the policy programs and plans [22]. In addition to the MoHME, the healthcare sector is being overseen and regulated through several medical professional bodies, with a non-governmental Iranian Medical Council (IMC) being the biggest among them [23]. The MoHME regulates and funds the provision and delivery of the primary healthcare services. The secondary and tertiary health care services are being financed through the public budget, out-of-pocket payments (OOP) and one of the four basic national health insurance funds (i.e., the SSO, the IHIO, the Armed Forces Medical Services Insurance Organization (AFMSIO) and Imam Khomeini Relief Foundation (IKRF)) [24, 25]. In addition to four basic insurance funds, 17 other smaller institutional funds provide health insurance coverage for their employees [26, 27]. These 17 funds are under the jurisdiction of the High Council for Health Insurance (HCHI) that is responsible for making changes to the social insurance provisions of each fund and sets the tariffs schedule for providers’ payments. The HCHI has members from the MoHME and Ministry of Labor, Cooperation and Social Affairs, as well as other stakeholders such as the IMC [28].

**Fig. 1:**
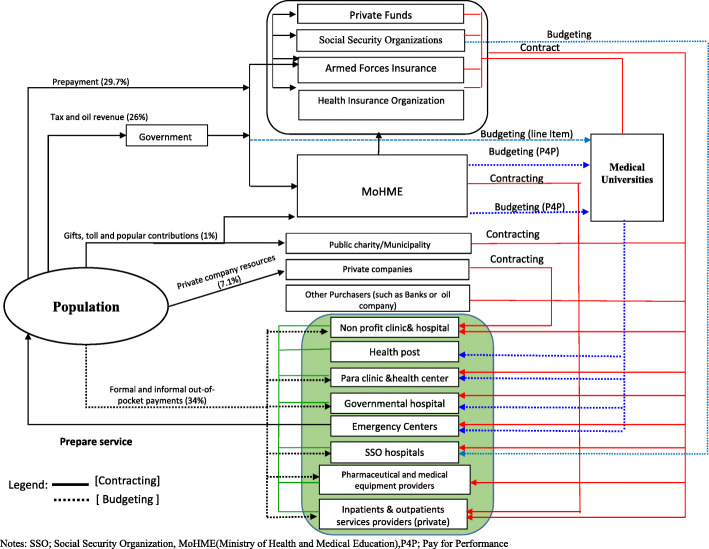
Financial streams within the Iranian healthcare sector

The existence of multiple and dispersed insurance funds and uncoordinated decision-making system for financing the health insurance organizations, the inefficacy of health financing schemes, duplication of coverage are the main challenges of the health insurance industry in Iran [29–31]. Currently, the health system funding in Iran comes from the government (23.8%), health insurance (30.6%), out-of-pocket payments (35.2%), private health insurance (6.1%), and individual donations and other sources (4.3%), based on data for 2018 [32]. Overall, the Iranian government currently spends ca. 8.4% of its GDP on healthcare. Figure 1 depicts the financial streams within the Iranian healthcare sector in 2019.

## Methods

We conducted documentary and literature reviews, stakeholder analysis, as well as qualitative interviews to reach a comprehensive understanding of the main historical policy-related time-events, trends, challenges, pitfalls, and drawbacks brought by the implementation of policy changes in medical tariffs setting process and explore possible solutions.

### Data collection

In the first phase of the study, we reviewed the official documents developed at the various levels of the Iranian health system, including the MoHME, insurance organizations, parliament health commission, and IMC. More specifically, we reviewed and analyzed the official policy documents, reports and regulations (such as five-year social, cultural, economic and political development plans), yearly published medical tariff booklets, and bylaws or the Iranian parliament’s proceedings that mentioned or discussed tariffs for different medical services during the various health ministries’ periods or presented empirical evidence related to changes in medical tariffs (Table 1). Unpublished documents were obtained in person. Additionally, we conducted a scoping review of relevant publications in several international (PubMed, Embase, ScienceDirect, Scopus) and the Iranian (Iran doc, Iran Medex, Scientific Information Database) databases using a combination of the following terms - Iran, medical tariffs, medical pricing, health and the Persian equivalents of the terms.

**Table 1:** Documents sources and Interviewees’ characteristics

Category	Number	Main samples
a) Documents sources and charactristics		
National Report, books, thesis, dessertation	19	Parliament’s Research Center reports, ‘Planning and Budget’ and ‘Health and Treatment’ Standing Committees reports, reports or books published by Social Security Organization Research Institute such as Reforms in Medical tariffs and 20-year setting medical tariffs in Iran, PhD or MSc thesis such as the effect of proposed changes of relative values of different specialists medical tariffs on payment weight of specialities and health system costs
Parliamentary Proceedings	123	Medical tariffs, medical services universal insurance, Hospital autonomy, hospital corporatization, Hospital self-sufficiency
Local Organizational websites	21	Parliament, Vice-presidency for Strategic Planning and Supervision, Medical Council, Medical Universities, Social Security Organization Research Institute, Major Public and Private hospitals, National centre for health insurance research
Acts, bills, proposals, bylaws and regulations,	91	The Organization and Duties of MoHME, Medical Services Universal Insurance, Rural Health Insurance, MoHME formation, Social Security, Public Financial Regulation, Hospital autonomy policy, First to Six Five-year Development Plans and related Acts, Reviewing system administration of hospital plan, Iran Medical Council formation, the Ministry of Welfare and Social Security, the Ministry of Cooperative, Labour and Social Welfare, Employee and contractual staff payment, Hospitals Boards of trustees, Health Transformation Plan, targeted subsidies plan
Academic literature	68	Papers published by the first seminar on assessing medical tariffs by Iran Medical Council, Papers published relevant to medical tariffs and related policies and reforms, mainly published in Farsi, English papers published in international journals
Others	8	Documents related to the working group as special representatives of the President on the duties of the government regarding health insurance
b) Interviewee characteristics		
Senior MoHME officials, senior management and planning organization deputies and officials, SSO officials, IMC senior policy makers, academic researchers, treatment deputy members		

*MoHME* Ministry of Health and Medical Organization, *SSO* Social Security Organization, *IMC* Iran Medical Council

To complement these reviews, we conducted 22 face-to-face interviews to reach data saturation. The semi-structured interview topic guide was based on the information acquired from the reviews. The interviewees were selected through purposive and snowball sampling methods. With one exception, all interviews were conducted at the participants’ place of work. We interviewed several stakeholders, including policymakers at the national level, health care services managers and officials, practitioners, and academics (Table 1). All interviews were audio-recorded and fully transcribed verbatim. The interviews lasted at a minimum of 80 min. The transcribed files were then sent back to the participants, allowing them to proofread the transcription and add or remove any information.

### Data analysis

We used a thematic framework approach to analyze data. Data analyses were performed in tandem with data collection. ‘Policy triangle’ [33] and the ‘garbage can’ models [34] were used to guide analyses and interpret the collected data. The ‘policy triangle’ model focuses on four inter-related aspects of the policy: context, process (agenda setting, formulation, implementation, and evaluation), content, and actors/stakeholders [33]. We used this model to show the complexity of historical changes in the development of the tariffs setting process in Iran. The ‘garbage can’ model views decision as an outcome of four interdependent streams (problems, solutions, participants, and choice opportunities) within the sector [34, 35]. We use it to outline the main shortfalls and drawbacks brought by the implementation of tariffs and describe underlying causes. Stakeholder analysis identified and the major stakeholders participating in the tariff setting. We used the four stages proposed by Mendelow [36]: determining whom the stakeholders are, rating the power of each stakeholder, rating the dynamism of each stakeholder, and allocating responsibility for scanning developments relating to each stakeholder group. Face-to-face interviews were analyzed using a thematic framework analysis approach and following five main steps, including familiarization, identifying a thematic framework, indexing, charting, and mapping and interpretation [37].

## Results

### Overview of changes in the Iranian tariff setting over the last half a century

We present our findings using four inter-related aspects of policy as subsections (context, process, content, and actors/stakeholders), following the ‘policy triangle’ model [33].

## Context

Before the national tariff system was created in 1972, budgetary payments were the only mechanism of paying to public hospitals and other public health services providers (Table 2). In 1972, following an extensive review of tariffs and prices in other countries with health insurance systems (i.e., Belgium, France, and the US), a first list of the tariffs was published and introduced into practice [38]. These tariffs remained unchanged for a decade until the first handbook of medical tariffs or ‘relative value units’ (called the ‘California Handbook’) was published in the US. In 1982, Iran adopted these tariffs by introducing a similar disease coding system, adjusting the relative value units, and applying the Rial coefficient (the K-factor), which would be revised annually based on the cost of living index to provide the actual and rational costs of medical services. However, ‘*due to confusion with the new disease coding system, the full-scale implementation of a tariff-based reimbursement system was delayed by three years, and most hospitals and physician practices continued to apply ‘old-style’ tariffs instead*’ [Doc 29, Majlis Report]. In 1985, the establishment of the MoHME invoked additional revision of tariffs for physician visits and hospital bed-day. In 1990, the health care tariffs were increased two-fold (compared to 1986 in relative terms) and remained unchanged for five years. In 1995, the Universal Medical Services Insurance (UMSI) Act declared that medical tariffs should be based on actual costs and be revised annually. After this Act, tariffs became the cornerstone in regulating the health care services market, financial autonomy of hospitals, and setting insurance premiums per capita [38].

**Table 2:** Key milestones in the establishment of the national tariff payment system (1972–1995)

Period	Milestone	Provider reimbursement	Controlled by
< 1972	1956: IMC created	Public: a line-item budget	Ministry of Work and Social Services
1972–1981	1972: first list of the tariffs	Public: a line item budget + tariffs-based reimbursement// Private: not clear	Ministry of Work and Social Services; SSO
1982–1985	1982: K-tariffs	idem + partially implemented new same tariffs for Private and public	Ministry of Work and Social Services; SSO
1985–1990	1985: MoHME created	idem + Introduced additional methods of reimbursement: K-tariffs + FFS + salary + capitation + bonus	MoHME
1990–1994	1990: UMSI Act introduced	idem, but the tariff is the primary method of reimbursement and shifts towards evidence-based tariff setting	MoHME, IMC
1995	1995: UMIO created	idem, tariffs are now revised annually based on total costs that are included. Return on invested capital and depreciation	MoHME

*IMC* Iran Medical Council, *SSO* Social Security Organization, *MoHME* Ministry of Health and Medical Organization, *FFS* Fee For Service, *UMSI* Universal Medical Services Insurance, *UMIO* Universal Medical Insurance Organization

## Process

Since 1995, the annual revision of the medical tariffs follows an established formalised process. First, a technical assessment of the annual costs is conducted independently by the MoHME and the insurance organisations, and occasionally by the IMC. The MoHME consults with other governmental agencies as well, such as Parliament Health Commission, Vice-presidency for Strategic Planning and Supervision of the MoHME, the Ministry of Cooperatives, Labour and Social Welfare (MCLSW), and special councils. Next, several technical meetings take place with representatives from the MoHME and insurance organisations to deliberate and agree on the tariffs incremental increase. Finally, agreed tariffs are presented to the HCHI for approval. Once approved by the HCHI and the Council of Ministers, tariffs are ready for implementation.

In theory, these steps should be completed before the start of a new fiscal year. In practice, however, this never happens [39]. In the last few years, the agreement was achieved as late as the second quarter of a new fiscal year. The tariff setting process is frequently halted by the private sector, large public hospitals, and medical universities that are usually lobbying for higher tariffs. ‘*Overall, despite occasional conflicts over health care services pricing, the private sector still works in close cooperation with agencies determining national medical tariffs*’ [Former senior policy officer]. At the same time, insurance companies impede the final agreement as well, since it allows to delay implementation and reimbursement using new higher tariffs.

For a new service to be added to the tariffs list, which is a prerequisite for it to being included in the insurance benefit package, it should first be approved by the HCHI. Such requests are usually initiated by hospitals or physicians. However, according to the participants, given the lack of corresponding medical tariffs that could act as a proxy for a new tariff, setting a tariff value for a new service is challenging. ‘*Coupled with significant delays due to negotiations between major stakeholders, lobbying efforts, insurers’ limited fiscal space for adding new services to the tariffs list, new services are rarely included in the insurance benefit packages*’ [Faculty member]. To overcome this obstacle, some of the speciality groups and hospitals set their tariffs via routes that sometimes do not involve the HCHI.

## Content

The tariffs are determined for hospital treatments and diagnostic services, medical inpatient care, laboratory and imaging services, and paraclinical services. These tariffs are split into three groups: outpatient doctor’s visits, FFS based on the ‘K rate’ of services, and hospital beds. The tariffs are also determined for some of the ambulatory care services, although by using a less sophisticated approach*.* Public provider tariffs are set at a lower rate as they are often being received monthly as a salary and rely on public sector infrastructure and staff to provide care.

### Dynamics of changes in medical tariffs during 1972–2017

**Fig. 2:**
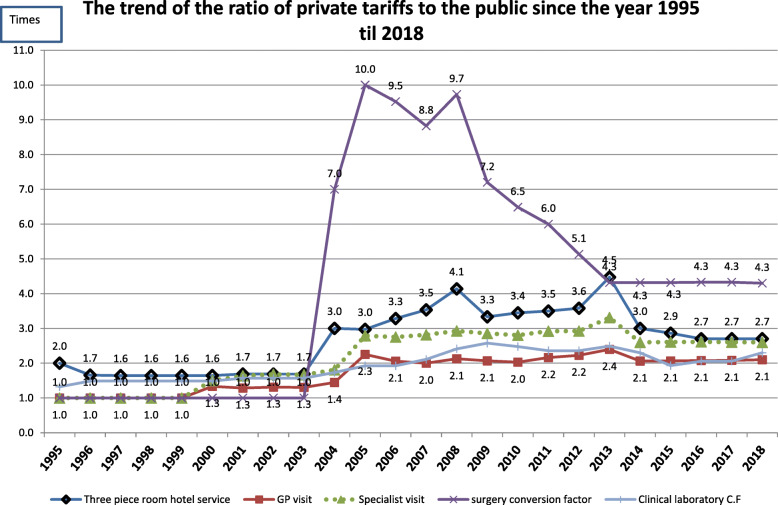
The trend of the ratio of private tariffs to the public since the year 1995 till 2018

**Fig. 3:**
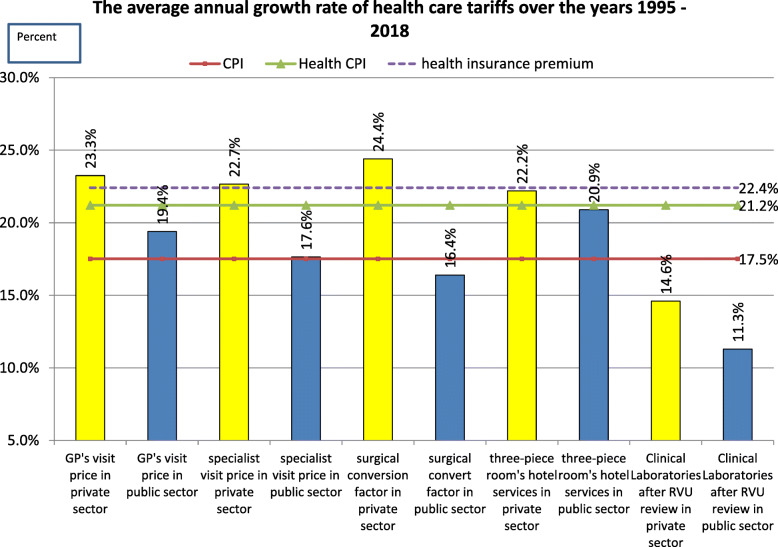
Growth rate of medical tariffs (1995 vs 2018)

From 1972 until 1992, the ratio between private and public tariffs remained fairly stable as both sectors had similar medical tariffs and insurance coverage. Medical tariffs were further split into public and private subgroups for laboratory, hoteling, and radiotherapy services in 1992, and for ambulatory (outpatient) physician visit subgroups in 2000 and inpatient services in 2003. Because of these splits, the gap between the public- and private-sector tariffs became more prominent (Figs. 2 and 3). Since 1995, by approving MSIA in Iran, tariffs were set to be revised annually, and there was an attempt to align them with the annual inflation rate [20]. Although the inconsistency between the inflation rate and medical tariffs remained to be a challenging issue in the country. In 2003, the IMC was allowed to set tariffs for private sector [40], contributing further towards increasing private/public tariffs ratio (particularly for internal medicine, anaesthesia and surgery services, up to 10 times more than public tariffs). ‘*The continuing imbalance resulted in dissatisfaction among different medical specialities and sometimes resulted in reducing the quality of health services or other outcomes in the health system, such as induced demand or overuse, the prevalence of informal payment, lack of transparency in the revenues and effect on the tax system and the country’s economic cycle, causing some health care providers avoiding signing a contract with insurance organisations*’ [Former advisor to the minister of health].

In 2014, MoHME implemented the Health Transformation Plan. The relative values were revised, and tariffs for public sector services were increased up to 2.2 times. After the reform, insurance organizations claimed that they could not reimburse all health care service to providers regularly [41]. ‘*Therefore, it can be concluded that along with the increase in the costs of med*i*cal services, an attempt has been made to subsidize insurance companies to fulfil their commitments, mainly those companies whose revenues do not depend on the salary of the insured*’ *[Oct 2016, Gazette No. 326].*

Overall, during the last half a century, tariffs for private health care services were consistently higher than those in the public sector (Fig. 2). However, the gap in tariffs fluctuated and was not consistent. Tariffs increased for all types of services to match the inflation rate (Fig. 3). In both public and private sectors, medical services costs had higher annual growth than inflation (except para-clinical services in both public and private sectors) [42]. For public services, the most substantial increase in tariffs was observed for hotel services in hospitals per diem and the lowest for clinical laboratory services (Fig. 3). For private services, the most substantial increase was for the relative unit-based services and the lowest for clinical laboratory services.

## Actors and stakeholders

The HCHI acts as a policy-making platform that facilitates the discussions and decisions surrounding key tariff-related issues, including insurance coverage, rate of insurance premium per capita and coinsurance, medical services costs, medical prices and supervision [24, 43]. However, its work is not without criticism. ‘*One of the major criticism regarding the HCHI is that individual council members, namely physicians, may have direct or indirect conflicts of interest and may affect the decisions made by the Council*’ [National policymaker]. The MoHME, HCHI, MCLSW, IMC, and the four basic health insurance organizations are the main actors in determining the tariffs. Most of these actors are governmental organizations.

Based on power and interest, we identified four main groups of stakeholders among eleven main actors (Table 3). Group 1 (high power and highly interested people) includes MoHME, IMC and basic health insurance organizations. They have more power and interest in defining tariffs than most. The MoHME, as the main actor in tariffs setting, should try to fully engage with other actors and make the most considerable effort to satisfy them. Group 2 (high power and less interested people) includes MCLSW, Parliament Health Commission and the Vice-Presidency for Strategic Planning and Supervision of the MoHME. MoHME should make enough effort to keep them satisfied, but not exhaust and bore them with the messages. Group 3 (low power and highly interested people) includes special councils and public/private hospitals. MoHME should try to adequately inform and engage them in discussion not to overlook any issues. Group 4 (low power, less interested people) includes 17 supplementary insurance funds and smaller stakeholders whose activity can be monitored but without priority and excessive communication.

**Table 3:** Role of stakeholders in the policy process of setting medical tariffs

	Actor	Role in setting medical tariffs	Activity Level	Activity Area	Position	Power	Influence	Agenda setting	Formulation	Implementation	Monitoring & evaluation	Group N
**Governance Side**	Parliament Health Commission	Approving macro policies such as five-year development plan and approving the basic health insurance yearly budgets for policy implementation.	National	Governmental	**–**	**High**	**–**	**+**	**+**	**+**	**+**	**2**
	Planning and budgetary organization	Approving proposed medical tariff revision and proposing to government, approving budget proposed by insurance organization, MoHME, and MCLSW.	National	Governmental	**+**	**High**	**Moderate**	**++**	**+**	**+**	**+**	**2**
	MoHME	Proposing policy of relative value revision and providing its implementation infrastructure.	National	Governmental	**+++**	**Very high**	**High**	**+++**	**+++**	**+++**	**++**	**1**
	MCLSW	Head of Insurance High Council and responsible for holding meetings and making related decisions.	National	Governmental	**+**	**High**	**High**	**++**	**++**	**++**	**+**	**2**
	Medical Council organization	Member of Insurance High Council, attendance in the meeting.	National	Non-Governmental	**+++**	**Very high**	**Very high**	**+++**	**+++**	**+++**	**+**	**3**
**Supply Side (Health care Providers)**	Private hospitals and clinics/para clinic	Health care provider and implementing and executing new tariff book.	Regional/local	Private	**++**	**Moderate**	**Very high**	**+**	**+**	**++**	**+**	**3**
	Public hospitals and clinics/ para clinic	Health care provider and implementing and executing new tariff book.	Regional/local	Governmental	**++**	**Moderate**	**Very high**	**+**	**+**	**++**	**+**	**3**
	Special Councils	Health care provider and implementing and executing new tariff book.	National/provincial	Governmental	**+++**	**Moderate**	**High**	**+++**	**+++**	**++**	**+**	**3**
**Demand Side (Health care purchasers or health caregiver)**	Basic Insurance organizations	Member of Insurance High Council, attendance in meetings and executer of tariff book.	National/provincial	Governmental	**+**	**Very high**	**–**	**++**	**++**	**++**	**++**	**1**
	Private/ supplementary insurance organizations	Member of the secretariat of Insurance High Council, attending in meeting and executer of tariffs.	National/provincial	Governmental	**–**	**Moderate**	**–**	**+**	**+**	**++**	**+**	**4**
	People/insured people	Health care givers and paying health care expenditures.	Regional/local	**–**	**–**	**Low**	**–**	**–**	**–**	**–**	**+**	**4**

*MoHME* Ministry of Health& Medical Education, *MCLSW* Ministry of Cooperatives, Labor, and Social Welfare; + − implies the participation role of the related organization in various stages of medical tariff setting from very strong (+++) to less strong (+)

### Major shortfalls and drawbacks brought by the implementation of medical prices and ways forward

#### Anarchy in medical tariffs system

‘Garbage can’ model assumes that policies are shaped and developed in an idiosyncratic way. It suggests that interventions that have been formally abandoned might survive in the system, solutions that have never been adopted may appear as legitimate policy options, and the policy options that were mean to be used in the system may disappear without attention of the decision-makers. The model, however, does not assume that no formal system exists; it rather suggests that these formal systems may behave chaotically alongside the informal arrangements. Such model presents policy-making as an untidy process rather than a neat series of phase [34, 35]. The ‘garbage can’ perspective can be useful when investigating the role of health system governance over time in setting and implementing tariffs in Iran. Some of the known characteristics of the Iranian health system are: a lack of a distinct stewardship mechanism in the tariff system, continuous disagreements among the stakeholders, lack of a transparent approach for the management of the conflicts of interests, a high turnover of organizational settings and their technical staff, and more importantly a lack of an objective and explicit mechanism for establishing and updating medical tariffs all may have played their role [44, 45]. Until now, the Iranian health system in the policy-making context does not have a unified and specific approach in policy regarding setting tariffs [41]. The existence of multiple organizations for decision-making has caused multiplicity in setting tariffs.

In our study, the ‘garbage can’ model illustrates that the organization and governance of tariffs setting consists of polymorphous patterns of different philosophies of health governance. According to some participants, ‘*.. even some so high-ranked policymakers were unclear about the main goal of the setting tariffs in Iran*’ [Senior policymaker]. Ironically, this ambiguity contributed to making tariff-related decisions regardless of implementation outcomes. ‘*This happened since tariffs increases proposed by the IMC were often not approved by the four basic health insurance funds, while they were readily implemented by the physicians in the private sector*’ [MoHME senior staff]. Political ideologies might also play a role in forming a ‘garbage can’ via pushing a topic to fore to demonstrate political dissatisfaction with the policy-making process [46]. While this might have played a role in Iran, especially after a period of presidential and parliamentary elections that resulted in different parties obtaining political power, we did not find clear evidence of such influence. Instead, we found that influential clinicians and clinical groups were pressuring politicians and policymakers to ensure the changes in the medical tariff system did not reduce their peers’ potential income. Tariff-based pricing of health services was a good starting point in Iranian health care financing. However, the improper adaptation of the tariffs, manipulating and involving some intentional changes in the relative values of healthcare services, caused moving tariff values away from the actual fiscal values of the health services. During the last years, irrational medical tariffs have caused some health professionals to request informal patients payments or attempt to get high revenues [47, 48]. The dissatisfaction of some stakeholders with tariffs has also produced different viewpoints about continuation or discontinuation of California book values as a reference point for tariff setting.

Analyzing interviews and documents showed that significant differences between medical tariffs in public and private sectors, as well as between intra- and inter-disciplinary tariffs, led to unfavourable outcomes listed below:

##### Elite students being propelled toward high-paying medical professions

Imbalance among relative values of tariffs for services of different medical specialities affected the delivery of health services and medical education system. Medical speciality residency programs in Iran select their candidates through an annual national exam, based on multiple-choice questions. Hence, students work hard to get higher marks and enter speciality routes with higher earning potentials. Such a situation resulted in the popularity of certain specialities with higher tariffs. ‘*Even among medical science graduates, there is a tendency to continue studies in high tariffs medical services or profitable fields, such that health care professionals are warning about the lack of interest in fields such as internal medicine and paediatrics and a greater interest in cardiology, ophthalmology, surgery, and radiology*’ [Health Researcher].

##### Development of the private sector for medical services and undermining of the public sector

With claims about unrealistic health services expenditure and the increased profit margin of medical services provided by the public sector, physicians are becoming more inclined to operate in the private sector: ‘*Moreover, the demand for less expensive services provided by private-sector institutions has increased, while resources, technologies, and management practices in the public sector have remained stagnant with the growth in demand*’ [Health insurance staff]. As a result, both patients and employees (physicians and non-physicians) get dissatisfied with the public sector. Legally, physicians are now prevented from simultaneously working in both public and private sectors (dual practice) [49, 50]. Legislators implied that the main reason for the tendency of physicians to leave the public sector or prefer to work in a private sector is the financial incentive, but failed to provide practical solutions to incentivize participation in the public sector [51, 52].

##### Governance power of actors in setting medical prices

Despite annually revised health care tariffs, there is no systematic costing process for health services, and the pricing system is still suffering from a lack of a transparent and balanced structure that can effectively manage conflicts of interest in decision making related to the medical services prices. Some experts believe that it is necessary to change actors’ roles in the tariff setting process. ‘*Unfortunately, during the last years public, non-public, private and semi-private organisations determine tariffs separately for their side and own benefits. They set tariffs based on individual agreements between their organisations and the insurance organisations or based on statutory authorities that sometimes resulted in unilateral increases in tariffs*’ [MoHME senior officer]. The highest authority in medical price setting (i.e., HCHI) suffers from an inappropriate membership composition. Its membership includes a heterogeneous group including insurance organizations representatives, the MOCLSW, the MoHME and the IMC. It seems that it is a time for the role of the MoHME in the pricing council to be more prominent. ‘*One of the main critics to the tariff setting system is that in tariffs context, there is no harmony between different decision-makers and groups that have more power have the main role in price setting and get more benefits*’ [Health insurance officer]. ‘*People’s expectation from governing actors who set prices and tariffs is to provide health services while upholding social equity, high quality of medical services and rational prices*’ [Medical Council officer].

Analysis of interviewees and documents showed that the organization and governance of medical tariffs setting consists of polymorphous patterns of different philosophies of health governance. Ironically, this ambiguity contributed to making tariff-related decisions regardless of implementation outcomes; for example, through implementing Health Transformation Plan and approving the medical tariffs systems within the MoHME before even ensuring that the main insurance organizations would support such changes. Another example is the transfer of the power of setting medical tariffs for the private sector to the IMC, which occurred in 2004 as part of the Five Year National Development Plan. Within the five years that this legislation was in power, it marked continuous challenges between the IMC and the insurers, rapid increases in the private sector tariffs, and increases in the share of out-of-pocket expenditure

##### Medical information systems and setting tariffs rationally

Despite improvements in the management of medical information systems in the hospitals, they still suffer from structural limitations that prevent detailed assessments of the health services costs. Most of the current information systems are developed based on the current pricing structure; hence, they are inadequate for assessing or modelling alternative approaches to provider payments. ‘*Determining the actual costs of the health services is an important input for revising and setting medical prices, but the limitations of the records and in the information system has meant that this has remained a challenge in Iran’s health care system*’ [A physician]. As a result, a provider that brings substantial revenue to the hospital might also produce substantial costs to the hospital because of material or human resources required for them. The latter costs, however, are not well-recorded in the system, and the hospital remains in the dark about the actual costs and benefits of the services. The limitation of the data at the local level reflects the problem at the national level where calculating and updating the relative values remains a challenge as it requires for micro-data to be available, while it is not. It becomes difficult to compare the actual costs of delivering services in different geographical regions or different settings.

##### Native model for health services tariff setting

Document analysis showed that, until now, the Iranian health system does not have a national health services tariff setting framework and evidence-based model. This issue should be addressed, as to achieve Universal Health Coverage, it is necessary to determine the actual price of health services based on scientific methods and new models. According to the interviewees determining the actual fiscal value of health services is also necessary to ensure equity in reimbursement of the costs to service providers in contrast to delivery and supply of these services. ‘*To balance the medical price market, it is necessary to set regulative (normative) tariffs that reflect the actual costs of service delivery and reliability in the development of health care delivery system and use appropriate mechanisms of setting health services tariffs*. Medical tariffs in public and private sectors need to be the same in order to increase the competition on increasing the quality of health care’ [Advisor to the minister of health]. Study participants also mentioned that periodic review of health care prices and revising them based on some indicators (e.g., health insurance per capita, inflation rate, and increasing index of the total cost of goods) is very important in setting those prices rationally as well.

## Discussion

The study explored the experience of setting medical tariffs in the Iranian healthcare system over the last half of the century. We discussed mechanisms for setting medical tariffs, its governance in the Iranian health system, and shared-decision making challenges, drawbacks and possible solutions.

Medical tariffs remain to be an increasingly debated issue in the field of health financing and medical payments in various countries. Tariffs belong to essential tools of policymakers that influence equity, efficiency, quality, responsiveness and accessibility to health services [11]. If utilised adequately and with the right support, medical tariffs have good potential to influence providers’ behaviour [53]. Governments can use tariffs to achieve their national policy goals and objectives. Through setting a rational tariffs system, governments can also provide an appropriate health financing management system.

Medical tariffs that were introduced as a policy tool in Iran became a tool for revenues manipulation in the country. Our findings imply that, unfortunately, during the last decades, Iran’s health system was continuously struggling with various problems and did not have a clear policy for the use of medical tariffs as leverage for policy-making. As a result, inappropriate medical tariffs setting and FFS payments led to higher volume and intensity of medical care (mainly due to induced demand), increasing health services costs, increasing out-of-pocket and households catastrophic payments, receiving under the table payments, and reducing patients’ satisfaction with Iranian health care system during the last decades [38, 52, 54, 55]. The process of setting tariffs annually has created a vicious circle. The cost of medical services should be determined by considering the insurers’ ability to pay or the annual premium rate, which is determined based on the payment capacity of the insured. Thus, people’s ability to pay is the most important constraint in determining the costs of medical services, which affects the tariffs of services. In the public sector, the difference in price rates is compensated through government subsidies, but in the private sector, this difference must be paid by individuals.

### Why intervene in medical tariff system?

The tariff system requires constant updating in response to new changes and innovations. As there are thousands of tariff codes, implementation is a complex process and calculation of the adequate payment rates is challenging. Such systems are also difficult to monitor and, hence, can become subject to abuse and fraud [56]. Also, if they are used in conjunction with the FFS payments, all negative characteristics of the FFS payment method need to be controlled [57]. On the positive side, because of linking service delivery to payments, such systems can generate data that can be used for assessment of a health system’s micro performance at the facility level and potentially by individual providers. All the challenges in health care system contribute to decision making based on individual, group or institutional interests and the dominance of bargaining power and non-technical views in the process of policy-making [33]. Regarding the unresolved issues in the medical tariffs setting system in the Iranian health care system, it can be argued that the current situation has rooted in a lack of accountability and transparency in decisions made in the medical tariffs system. Although there seem to be equity and quality concerns over the continuation of the current system that has developed in more than four decades, the challenges in the Iranian health system context does not allow establishing appropriate payment mechanisms and financing in the health care system.

The findings of this study suggest that major problems in the Iranian health system are due to flawed medical services tariff setting systems, which in turn are caused by underlying factors such as lack of transparency, conflicts of interest, incorrect pricing of medical services, and the complex nature of the health care system. Therefore, due to inelasticity of medical services costs and the pressure on consumers to pay the medical services costs, insurance coverage must be expanded in a way to reduce the household expenditure and remove the direct payment between patients and healthcare providers [3, 4, 58]. Our findings also suggest the need for the payment system reform in Iran by evaluating distortions such as length of stay, use of health care facilities and services, and overall health care costs in different levels of the health system that the FFS reimbursement has induced. It seems to transition from FFS reimbursement to the diagnostic related groups-based prospective payment system for inpatient care, or other prospective payment systems should be a priority of health policymakers in Iran. However, establishing any forms of provider payment requires robust administrative and appropriate services delivery infrastructure.

Tariffs setting based on an optimal payment system that involves mixed levels of both demand-side and supply-side cost-sharing is the main step in rationalizing health services. It is widely believed that financial incentives of health care providers affect their care delivery behaviour and efficiency of health care [59–61]. Any change in the medical tariffs should be determined using evidence-based and transparent criteria, imposed by fair pricing laws to ensure providers are given the right motivations and incentives for effective delivery of services [59]. Also in setting tariffs, the input costs of the services (including physical standards and expertise), the complexity of the services and the time required, risk of adverse outcomes, long term follow up requirements, geographical location and setting of care are among other factors that should be considered [10].

Using healthcare tariffs for financing healthcare is an important policy decision. It has a vast influence on behaviours of health providers and users of health services, and it may determine the accessibility, service coverage, and equity and efficacy objectives [35]. Although tariffs are one of the ways for financing, it should not be the primary means of financing, and should not be applied uniformly; else, the wealthy will benefit, and the poor will suffer [23]. After the Health Transformation Plan reform, specialist medical prices increased significantly. It needs to be considered that even a significant increase in funding in a health system will not be enough unless a country has an appropriate organisation and infrastructure for the effective use of all types of resources [62]. Failing to observe a trade-off between the fee-changes and efficiency gains in health care may be surprising. Our results generally imply that providers alter their care behaviours in response to medical price changes in ways that can have an impact on patient outcomes, similar to previous studies [60].

### Limitations and strengths

In the process of data collection, access to some documents was not possible. For example, the provisions of approved directives and circulars relative to the medical tariffs before 1995 are not in access or reliable. As such, we could not identify the exact separation time of medical tariffs, such as costs of hospitalization (hoteling costs) or medical laboratory and medical imaging services for both the public and private sectors. We could not zoom our study into the micro-level and explore or focus in great detail changes in volumes/shares of finances and how it affected interconnected elements of the health system. In this study, we also did not use dynamic modelling. However, we managed to complement our findings from literature with face to face interviews that reflected these micro-level views. To our knowledge, this is the first study on medical tariffs setting that comprehensively explored the historical trend of medical tariffs setting, influential factors, challenges, causes, and solutions. More studies are needed, and we recommend proper before and after policy evaluation and ongoing monitoring of any reforms.

## Conclusion

Setting fair and justified tariffs for health care services is a complex task given interconnectedness and complexity of major stakeholders’ relationships, cultural aspects, and the legacy of laws. More definite plans and strategy, stricter division of the roles in power matrix, with delineating roles and funding streams, revision of the insurance plans are needed to have a productive way forward. Medical tariffs policy in Iran has substantially changed over the last half of a decade and consequentially has had a substantial impact on most critical functions of the health system, including health care providers’ behaviour, payments, organization, regulation, and financing. To inform the policy debate, this paper profiled experiences (challenges, causes and solutions) of the Iranian health care system on the setting tariffs of health care services. Evidence should be used for any efforts to rectify the medical tariffs system in Iran.

Many of the challenges and problems in setting medical tariffs relate to political governance, power and surveillance, structural organization of medical tariff system, methods, and principles of setting tariffs, medical costs recording systems and conflict of interest in the medical tariff system. To improve medical tariffs system in the country, one needs to have a deep understanding of the current challenges and potential solutions at different levels of the health system. Overall, the creation of national tariffs setting framework and application of scientific methodology and methods to the decision analysis in setting medical tariffs is necessary to ensure improvement in health sector performance.

## Data Availability

All data are available from the corresponding author upon reasonable request.

## Abbreviations

MoHME: Ministry of Health and Medical Education
FFS: Fee-for-service
SSO: Social Security Organization
IHIO: Iran Health Insurance Organization
IMC: Iranian Medical Council
OOP: Out-of-pocket payments
AFMSIO: Armed Forces Medical Services Insurance Organization
IKRF: Imam Khomeini Relief Foundation
HCHI: High Council for Health Insurance
UMSI: Universal Medical Services Insurance
MCLSW: Ministry of Cooperatives, Labour and Social Welfare
